# The impact of codeine rescheduling on non-opioid analgesic use by people who regularly use codeine: a prospective cohort study

**DOI:** 10.1007/s11096-024-01751-9

**Published:** 2024-07-15

**Authors:** Jessie Maher, Jacqui McCoy, Raimondo Bruno, Suzanne Nielsen

**Affiliations:** 1 School of Medicine, The University of Notre Dame Sydney, Chippendale, Australia; 2 School of Psychology, The University of Tasmania, Sydney, Australia; 3 Monash Addiction Research Centre, Eastern Health Clinical School, Monash University, Peninsula Campus, Rm 205A, Level, 2, Building D, 47-49 Moorooduc Hwy, Frankston, VIC 3199 Australia

**Keywords:** Analgesic, Codeine, Opioid, Pain, Policy, Rescheduling

## Abstract

**Background:**

Codeine was rescheduled in Australia to prescription only in February 2018. Initial studies reported an increase in population level paracetamol and ibuprofen sales following codeine upscheduling. However, to date no study has been able to investigate changes in non-opioid analgesic use at the individual patient level to determine if sales data reflect actual consumption patterns.

**Aim:**

To address this gap, we aimed to determine the impact of codeine rescheduling on non-opioid analgesic use in people who regularly used over-the-counter codeine, primarily for pain, prior to the rescheduling change.

**Method:**

We conducted a prospective cohort study with 260 participants who reported regular over-the-counter codeine consumption at cohort entry. Surveys were completed at baseline (November 2017, 3 months before rescheduling) and at 1 month (February 2018), 4 months (June 2018), and 12 months (February 2019), following rescheduling. The primary outcomes were mean daily doses of non-opioid analgesics, captured through a 7 day medication diary.

**Results:**

The mean daily paracetamol dose decreased from 1754.4 mg (95% CI 1300.5–2208.3) at baseline to 1023.8 mg (95% CI 808.5–1239.1) at the final time-point (+ 12 months) (*p* = .009). The mean daily ibuprofen dose decreased from 305.1mg (95% CI 217.9–392.4) at baseline to 161.2 mg (95% CI 98.5–224.0) 12 months after rescheduling (*p* = .03). No significant change in doses of other medications remained was found.

**Conclusion:**

In people who regularly consumed over-the-counter codeine, doses of non-opioid analgesics either reduced or remained stable following codeine rescheduling, suggesting concerns of medication substitution or overuse following the change were not realised.

## Impact statements

- Upscheduling of medicines is a key policy strategy used to address harm with pharmaceutical drugs.
- In contrast to sales data which suggested increased paracetamol and ibuprofen purchasing following the rescheduling of codeine, our findings showed doses of paracetamol and ibuprofen at 12 months following the changes were significantly lower than at baseline.
- In people who regularly consumed codeine, doses of non-opioid analgesics either reduced or remained stable following codeine rescheduling.
- Codeine rescheduling did not appear to drive increased use of simple analgesics.

## Introduction

In response to concerns about harm resulting from inappropriate use, and continued evidence of harms, medications containing low dose codeine were restricted in Australia to become prescription-only medicines in February 2018 [1]. A number of other countries are considering similar changes, making studies that document outcomes of this change critical for policy makers internationally. A systematic review of rescheduling hydrocodone in the US found that the outcomes were mixed, with some reductions in hydrocodone use but an increase in prescribing of other opioids [2]. A second global review of the evidence of up-scheduling medicines found that while up-scheduling can reduce use of the unscheduled medicine, substitution effects with increases in other substance use were common [3]. A small number of studies have investigated the outcomes of the Australian 2018 codeine rescheduling on population level outcomes. The number of low dose codeine medications purchased in Australia decreased by 87% [4]. Calls to the New South Wales Poisons Information Centre related to codeine also decreased by 50% [4]. Hospital attendances for codeine poisoning decreased by 53% [5]. At the same time, prescriptions for codeine and other opioids, remained relatively stable [6].

Although pharmacists believe codeine rescheduling would benefit their patients [7, 8] few studies have directly investigated the impact of codeine rescheduling on individual patients. One study surveyed Australian adults perceptions, finding mixed views on the rescheduling decision [8]. A prospective cohort examined the impact the removal of OTC codeine sales on codeine use and other health outcomes in people who frequently used OTC codeine prior to the scheduling change [9]. This study demonstrated a significant reduction in codeine consumption after the policy change [9]. However, the impact of codeine rescheduling on the use of non-opioid analgesics, including paracetamol and non-steroidal anti-inflammatory drugs (NSAIDs) in people who were using over-the-counter codeine is unknown. Sales of paracetamol and ibuprofen increased after rescheduling, suggesting the potential for increased reliance on alternative non-opioid analgesics [10]. As overuse of these medications can cause a range of complications, including liver failure and peptic ulcers, it is important to examine this as an outcome [10], so any potentially harmful trends in the use of non-opioid analgesics are identified.

### Aim

This study aimed to determine the impact of the 2018 codeine rescheduling on non-opioid analgesic use in a cohort of people who regularly used OTC codeine in Australia.

### Ethics approval

This study received ethics approval on November 3, 2017 from the Tasmanian Health and Medical Human Research Ethics Committee, reference number H0016685.

## Method

The methods were described in the prospectively published protocol [11]. All methods relevant to the current analyses are also described below, and the study has been reported as per the STROBE statement [12].

### Study design and setting

We conducted a prospective cohort study with online questionnaires completed at baseline and at several times following codeine rescheduling, which occurred on February 1, 2018. Time-points were baseline (November 2017, 3 months before) and 1 month (February 2018), 4 months (June 2018), and 12 months (February 2019), following rescheduling.

### Participants

Inclusion criteria for this study were that participants had to be at least 18 years old, with regular codeine use in the preceding 6 months. To assess regularity of codeine use, screening questions about OTC use asked respondents to indicate if codeine was used ‘every day’, ‘a few times week, ‘once a week’, ‘a few times a month, ‘at least monthly’ or ‘less than monthly’. Regular use was defined as consuming codeine ‘at least a few times per week’ or ‘every day’. People who were receiving treatment for codeine dependence were excluded. There were 260 participants at baseline, 204 participants at t1 (+ 1 month), 188 participants at t2 (+ 4 months) and 174 participants at t3 (+ 12 months).

### Recruitment

Various online methods were used to recruit participants, including posts on national health websites, university pages and social media (Facebook and Twitter). Emails were also disseminated to participants from earlier codeine research [13]. An online screening survey was used to determine eligibility. Following this, participants who met the inclusion criteria were sent the online baseline questionnaire (− 3 months, November 2017). All participants who completed the baseline questionnaire were sent the follow-up questionnaires. Participants went in the running to receive a $100 gift voucher for completing the baseline survey and received a $20 gift voucher when completing each time-point after this. Participants were contacted at follow-up was via email, text, and phone call with reminders to complete the survey.

### Measures

#### Non-opioid pain medicines

A list of non-opioid pain medications were selected using references including the therapeutic guidelines (Pain and analgesia) and relevant studies [10, 14, 15]. Medications of interest, which were each examined individually, included paracetamol, aspirin and NSAIDs (ibuprofen, celecoxib, naproxen, diclofenac, indomethacin, meloxicam) [10, 14, 15]. Various adjuvants commonly used for neuropathic pain were also included, such as anticonvulsants (gabapentin, pregabalin), tricyclic antidepressants (amitriptyline, nortriptyline), and serotonin noradrenaline reuptake inhibitors (duloxetine, venlafaxine, desvenlafaxine) [14]. Of these medications, indomethacin and nortriptyline were not included in the analyses, due to minimal usage across all time-points.

Medications of interest were coded using the Anatomical Therapeutic Chemical (ATC) classification system [15]. Medication strength and dose were reviewed for accuracy, using pharmaceutical resources [16]. Corrections were applied where appropriate, for example where participants entered the codeine strength (various) instead of the paracetamol strength of 500 mg, for combination medications (as codeine was the focus of the original parent study) [9].

### Primary outcome

The primary outcome for the current study was the mean daily dose of non-opioid analgesics collected using a one-week medication diary. This was calculated by adding each participant’s total weekly dose for a specific medication and dividing the weekly total by seven [9]. The total was divided by the number of participants at each time-point, to calculate the mean daily dose [9]. 

### Analysis

IBM SPSS Statistics 28.0.1 software was used for statistical analysis. Mixed-effects regression models were used to analyse data for the primary outcome of mean daily dose of non-opioid analgesics (mg, continuous variable), to determine if doses changed significantly with time. Mixed-effects models account for both fixed (time) and random effects (participants) [17]. A diagonal covariance structure was used. Analyses were conducted using all participant data. A complete case analysis (individuals who completed all time-points) was conducted as a sensitivity analysis.

We examined the frequency of non-opioid analgesic medication use at each time-points. Participants at every time-interval were included as the main results. We estimated marginal means and 95% CI for mean daily doses. *F*, *P* values and Cohen* d* are also recorded, where the estimated marginal means at baseline were compared with that of the final time-point (+ 12 months). Analyses also determined the median daily dose and IQR, amongst participants who took the medication of interest only where at least 15 participants had taken the medication at each time point. This enabled a comparison between the median dose amongst those taking the medication and mean dose for all participants, given that the mean dose calculations may have been artificially lower if fewer participants consumed that medication at a given time-point. Mixed models analyses using fully standardised data produced results consistent with the raw data. The latter is retained in the manuscript for ease of interpretation. Hypotheses were not pre-registered and therefore the results should be considered exploratory.

## Results

### Participant demographics

Participants at baseline were a mean of 39 years (*SD* = 13.6). Most participants (60%, n = 156/260) were female, most (81%, n = 170/209, measured at February 2018) had completed year 12 or further tertiary education), and almost all (99%, 258/260) resided in stable housing.

### Non-opioid analgesic use

Paracetamol was the most common non-opioid analgesic consumed by participants at all time-points (Table 1). At baseline, 69% (169/244) of participants reported paracetamol use. Ibuprofen was the next most frequently consumed analgesic, with 32% (79/244) of participants consuming it at baseline. Over time, the proportion of participants taking non-opioid analgesics remained relatively stable.

**Table 1:** Frequency of participants taking non-opioid analgesics: all participants

Outcomes	t = − 3 months n = 244 *n* (%)^a^	t = + 1 month n = 204 *n* (%)^a^	t = + 4 months n = 190 *n* (%)^a^	t = + 12 months n = 175 *n* (%)^a^
Paracetamol	169 (69)	115 (56)	107 (56)	101 (58)
Ibuprofen	79 (32)	50 (25)	45 (24)	37 (21)
Aspirin	8 (3)	4 (2)	3 (2)	6 (3)
Celecoxib	7 (3)	6 (3)	5 (3)	7 (4)
Naproxen	9 (4)	7 (3)	6 (3)	6 (3)
Diclofenac	7 (3)	8 (4)	4 (2)	5 (3)
Meloxicam	9 (4)	6 (3)	9 (5)	5 (3)
Gabapentin	8 (3)	7 (3)	3 (2)	8 (5)
Pregabalin	26 (11)	21 (10)	20 (11)	17 (10)
Amitriptyline	15 (6)	15 (7)	14 (7)	15 (9)
Duloxetine	10 (4)	6 (3)	8 (4)	11 (6)
Venlafaxine	17 (7)	11 (5)	11 (6)	15 (9)
Desvenlafaxine	8 (3)	6 (3)	6 (3)	8 (5)

^a^To calculate the percentage of *n*, the following sample sizes were used. n = 244 (16 missing medication entries) at baseline (t = − 3 months), n = 204 (56 missing medication entries) at February 2018 (t =  + 1 month), n = 190 (70 missing medication entries) at June 2018 (t =  + 4 months), and n = 175 (85 missing medication entries) at February 2019 (t =  + 12 months)

### Mean daily dose of non-opioid analgesics

The mean daily dose of paracetamol for the whole cohort (including those that reported no medication use) decreased over time, from 1754.4 mg (95% CI 1300.5–2208.3) at baseline to 1023.8 mg (95% CI 808.5–1239.1) at the final time-point (+ 12 months), with a small magnitude effect size (*P* = 0.009, Cohen *d* = 0.2) (Table 2). The mean daily ibuprofen dose also decreased from 305.1 mg (95% CI 217.9–392.4) at baseline to 161.2 mg (95% CI 98.5–224.0) 12 months after codeine rescheduling (*P* = 0.03). There was no significant overall effect of time on mean daily dose for the remaining medications (Tables 2 and 3). No analgesic examined had increased use over time.

**Table 2:** Mean daily dose of non-opioid analgesics: all participants

Outcomes	*F*	*P* value	*n*^a^	Cohen *d*^b^	t = − 3 Months mean (95%CI)^c^	t = + 1 Month mean (95%CI)^c^	t = + 4 Months mean (95%CI)^c^	t = + 12 Months mean (95%CI)^c^
Paracetamol (mg)	F_315_ = 3.93	.009	256	− 0.23 [− 0.448, − 0.016]	1754.41 (1300.50, 2208.32)^R^	1110.50 (885.40, 1335.60)^*^	952.72 (747.62, 1157.81)^**^	1023.79 (808.53, 1239.05)^*^
Ibuprofen (mg)	F_318_ = 3.00	.031	256	− 0.18 [− 0.399, 0.034]	305.14 (217.89, 392.38)^R^	204.72 (128.86, 280.58)	168.95 (105.21, 232.68)^*^	161.23 (98.50, 223.97)^*^
Aspirin (mg)	F_417_ = 2.19	.089	256	− 0.09 [− 0.301, 0.131]	20.19 (4.00, 36.38)^R^	16.80 (− 3.65, 37.24)	2.64 (− 0.72, 6.00)^*^	6.89 (− 1.52, 15.31)
Celecoxib (mg)	F_216_ = 0.30	.825	256	0.06 [− 0.157, 0.274]	5.39 (− 0.05, 10.83)^R^	5.73 (− 0.29, 11.75)	4.97 (0.35, 9.59)	7.10 (1.90, 12.31)
Naproxen (mg)	F_236_ = 0.33	.801	256	0.02 [− 0.194, 0.237]	24.62 (5.85, 43.39)^R^	34.44 (9.91, 58.97)	26.50 (8.52, 44.49)	26.68 (4.31, 49.05)
Diclofenac (mg)	F_215_ = 0.90	.442	256	0.05 [− 0.169, 0.272]	1.98 (0.33, 3.63)^R^	7.93 (− 2.03, 17.90)	1.43 (− 0.18, 3.04)	11.36 (− 8.07, 30.79)
Meloxicam (mg)	F_207_ = 0.20	.896	256	− 0.04 [− 0.252, 0.18]	0.68 (0.14, 1.22)^R^	0.55 (− 0.05, 1.15)	0.63 (0.07,1.19)	0.57 (− 0.05, 1.19)
*Gabapentin (mg)*^*d*^								
Pregabalin (mg)	F_295_ = 0.92	.432	256	0.11 [0.111, 0.321]	24.44 (13.49, 35.39)^R^	39.58 (8.01, 71.15)	27.93 (15.62, 40.24)	38.13 (16.80, 59.46)
Amitriptyline (mg)	F_128_ = 0.04	.991	256	0.02 [− 0.216, 0.249]	2.70 (0.78, 4.62)^R^	2.59 (0.69, 4.48)	2.71 (0.42, 5.00)	2.85 (0.36, 5.34)
Duloxetine (mg)	F_194_ = 1.16	.325	256	0.15 [− 0.069, 0.363]	3.05 (0.73, 5.37)^R^	2.61 (0.53, 4.70)	3.33 (1.33, 5.33)	4.06 (1.29, 6.84)
*Venlafaxine (mg)*^*d*^								
Desvenlafaxine (mg)	F_195_ = 0.90	.441	256	0.04 [− 0.177, 0.255]	4.73 (0.88, 8.58)^R^	6.64 (0.36, 12.93)	3.90 (− 0.12, 7.92)	5.84 (− 0.08, 11.75)

^a^The sample size was *N* = 260 at baseline (t = − 3 months) and *n* = 174 at February 2019 (t =  + 12 months). The reported *n* may be lower than 260 because some participants completed the survey but did not complete the medication diary

^b^Cohen *d* effect sizes involve comparison of baseline (November 2017) and 12 month follow-up (February 2019) time points. Effect sizes were calculated manually using Estimated Marginal Means. Sample size in these calculations was the *n* at t =  + 12 months, as this was the lowest *n*

^c^Mean (95%CI) refers to the Estimated Marginal Means and the associated 95% Confidence Interval and were used for continous variables

^d^The mixed model analysis for gabapentin and venlafaxine did not run, likely due to low sample size at various time-points

^R^Refers to the reference group for pairwise comparisons of Estimated Marginal Means

^*^P < .05

^**^P < .001

**Table 3:** Mean daily dose of non-opioid analgesics: complete cases (participants who completed all time-points)

Outcomes	*F*	*P* value	*n*^a^	Cohen *d*^b^	t = − 3 Months mean (95%CI)^c^	t = + 1 Month mean (95%CI)^c^	t = + 4 Months mean (95%CI)^c^	t = + 12 Months mean (95%CI)^c^
Paracetamol (mg)	F_237_ = 3.94	.009	142	− 0.25 [− 0.48, − 0.013]	1592.97 (1226.70, 1959.23)^R^	1128.63 (856.02, 1401.23)^*^	973.44 (723.79, 1223.09)^*^	993.71 (760.09, 1227.34)^*^
Ibuprofen (mg)	F_232_ = 3.36	.020	142	− 0.21 [− 0.441, 0.026]	274.75 (175.41, 374.09)^R^	198.44 (106.54, 290.34)	171.23 (93.07, 249.38)	120.12 (64.94, 175.30)^*^
Aspirin (mg)	F_308_ = 1.03	.379	142	− 0.06 [− 0.294, 0.171]	19.32 (− 2.38, 41.01)^R^	12.07 (− 5.31, 29.46)	3.52 (− 0.99, 8.03)	7.63 (− 2.62, 17.88)
Celecoxib (mg)	F_152_ = 0.79	.499	142	0.12 [− 0.108, 0.357]	5.33 (− 0.43, 11.10)^R^	8.05 (0.42, 15.68)	7.55 (0.75, 14.34)	9.56 (2.25, 16.86)
Naproxen (mg)	F_218_ = 0.37	.777	142	0.12 [− 0.118, 0.348]	24.80 (− 1.88, 51.47)^R^	29.93 (− 2.68, 62.53)	27.92 (2.73, 53.11)	33.95 (5.80, 62.10)
Diclofenac (mg)	F_165_ = 0.92	.431	142	0.05 [− 0.183, 0.291]	2.69 (0.29, 5.09)^R^	2.82 (− 0.48, 6.11)	1.16 (− 0.59, 2.90)	13.98 (− 10.01, 37.98)
Meloxicam (mg)	F_162_ = 0.19	.901	142	− 0.05 [− 0.285, 0.18]	0.70 (0.17, 1.24)^R^	0.58 (− 0.05, 1.21)	0.57 (0.04, 1.11)	0.55 (− 0.08, 1.17)
*Gabapentin (mg)*^*d*^								
Pregabalin (mg)	F_180_ = 2.12	.099	142	0.11 [− 0.127, 0.34]	11.62 (2.80, 20.44)^R^	18.49 (6.29, 30.68)	22.54 (10.10, 34.97)	30.11 (8.00, 52.21)
Amitriptyline (mg)	F_162_ = 0.07	.975	142	0.02 [− 0.215, 0.25]	3.94 (0.39, 7.50)^R^	3.73 (0.31, 7.16)	3.84 (0.89, 6.79)	4.16 (1.09, 7.22)
Duloxetine (mg)	F_143_ = 1.06	.370	142	0.13 [− 0.105, 0.361]	3.38 (0.04, 6.72)^R^	2.96 (0.06, 5.85)	3.87 (1.12, 6.63)	4.51 (0.93, 8.08)
*Venlafaxine (mg)*^*d*^								
Desvenlafaxine (mg)	F_148_ = 0.90	.445	142	0.04 [0.195, 0.27]	5.99 (− 0.26, 12.23)^R^	8.80 (− 0.60, 18.21)	4.93 (− 1.54, 11.40)	7.39 (− 0.95, 15.74)

^a^The sample size was *N* = 260 at baseline (t = − 3 months) and *n* = 174 at February 2019 (t =  + 12 months). The reported *n* is lower because this analysis included complete cases only

^b^Cohen *d* effect sizes involve comparison of baseline (November 2017) and 12 month follow-up (February 2019) time points. Effect sizes were calculated manually using Estimated Marginal Means. Sample size in these calculations was the *n* at t =  + 12 months, as this was the lowest *n*

^c^Mean (95%CI) refers to the Estimated Marginal Means and the associated 95% Confidence Interval and were used for continous variables

^d^The mixed model analysis for gabapentin and venlafaxine did not run, likely due to low sample size at various time-points

^R^Refers to the reference group for pairwise comparisons of Estimated Marginal Means

^*^P < .05

^*^P < .001

Similar results were seen in the complete case analysis, which was limited to participants who completed medication diaries at all four time-points (Table 3). The mean daily dose of paracetamol decreased significantly, from 1593.0 mg (95% CI 1226.7–1959.2) at baseline to 993.7 mg (95% CI 760.1–1227.3) at the final time-point, with a small magnitude effect size (*P* = 0.009, Cohen *d* = 0.3). The mean ibuprofen dose decreased from 274.8 mg (95% CI 175.4–374.1) at baseline to 120.1 mg (95% CI 64.9–175.3) at 12 month follow-up, and this was statistically significant (*P* = 0.02). There was no significant overall effect of time on mean daily dose for any other medication.

### Median daily dose of non-opioid analgesics

We determined the median daily dose amongst participants who took the medication of interest only (as opposed to across the whole cohort) (Table 4). The median daily dose of paracetamol decreased from 1714.3 mg (IQR 2393) at baseline to 1142.9 mg (IQR 2571) 12 months after codeine rescheduling. The median daily dose of ibuprofen amongst participants who consumed it, was 800.0 mg (IQR 857) at baseline. Although the median dose decreased initially (t =  + 4 months), it returned to 800.0 mg (IQR 971) at the final time-point. The median dose of pregabalin and amitriptyline remained relatively stable.

**Table 4:** Median daily dose of non-opioid analgesics: participants who took medication of interest  (with at least 15 participants per time point)

Outcomes	t = − 3 months		t = + 1 month		t = + 4 months		t = + 12 months	
	n	Median (IQR)	n	Median (IQR)	n	Median (IQR)	n	Median (IQR)
Paracetamol (mg)	169	1714.30 (2393)	115	1142.90 (3490)	107	1000.00 (1571)	101	1142.90 (2571)
Ibuprofen (mg)	79	800.00 (857)	50	800.00 (971)	45	457.10 (1029)	37	800.00 (971)
Pregabalin (mg)	169	200.00 (150)	26	160.00 (213)	21	175.00 (150)	17	225.00 (388)
Amitriptyline (mg)	79	25.00 (30)	15	20.00 (30)	15	25.00 (60)	15	25.00 (30)

## Discussion

### Statement of key findings

We aimed to determine the impact of codeine rescheduling on non-opioid analgesic use in a cohort of people who regularly used OTC codeine prior to rescheduling. We did not observe an increase in the doses consumed of any non-opioid analgesics examined following the removal of OTC codeine from sale. This remained the case where mean doses were calculated for the whole cohort and when the analysis was limited to those taking the analgesic of interest. For paracetamol and ibuprofen, the mean dose decreased significantly after codeine rescheduling. Doses of pregabalin and amitriptyline remained relatively stable. We found was no evidence of substitution of OTC codeine with alternative non-opioid pain medications, or overuse of non-opioid medicines.

The decline in mean dose of paracetamol and ibuprofen observed in this cohort is contrary to findings from initial population level studies where sales of common analgesics increased following codeine upscheduling [10]. A potential explanation for the decreased overall consumption of the analgesics observed in this study is that codeine combination analgesics also included these ingredients so once these medications became prescription only, their decreased use would have subsequently reduced the total amount of paracetamol and ibuprofen consumed. It appears that this was not offset by these products being taken as single ingredient preparations. Alternatively, population level sales of ibuprofen and paracetamol in sales may not reflect consumption as purchased analgesics may not be consumed. Further, increased general practitioner (GP) consultations were observed following rescheduling, alongside a small but statistically significant (although not clinically meaningful, representing approximately half a point on a ten point scale) reduction in pain scores [9]. These GP consults may have included alternative pain management support which may explain why additional non-opioid analgesia was not required.

### Strengths and weaknesses

A strength of this study is prospective cohort design where detailed medication information was collected at the individual level from the target population of the intervention. A further strength is that doses of non-opioid analgesics were calculated both for the whole cohort and for only those taking each medication at each time point. Limitations of this study includes the potential for recall bias. A one week medication diary with a short recall periods is a common method used to collect medication data in prospective studies to minimise this concern [18]. Although there was attrition of the original cohort, a considerable proportion of the original sample remained at the final time-point (174 participants), with a retention rate of 67% [9]. The mean dose analyses were comparable for all the whole sample and the complete cases analysis, providing reassurance that attrition did not significantly affect study results. There is the possibility that the sample were affected by selection bias due to online nature of recruitment, however we note that there are very high rates of access to internet and smartphone access in Australia, covering more than 95% of the population [19]. Finally, we only followed participants for 12 months after the change.

### Interpretation

Neither this study, or findings of previous studies, have found evidence of unintended outcomes from codeine rescheduling at the individual or population level [9]. This has important implications for other countries considering codeine rescheduling. Codeine rescheduling appears to have reduced codeine use and associated harms without substitution to other opioids or overuse of alternative non-opioid analgesics [9]. When taken together, the results of these studies should provide reassurance to healthcare workers and policy makers, that complications of non-opioid analgesic misuse, including liver failure and peptic ulcers, are not likely to have occurred as a direct result of the change [10, 20].

### Further studies

Further studies with larger sample sizes may examine the longer-term outcomes of codeine upscheduling on medication use.

## Conclusion

In conclusion, we found codeine rescheduling was not associated with increased use of non-opioid analgesics, providing further evidence that upscheduling did not appear to have unintended outcomes.
